# Control Analysis of Protein-Protein Interaction Network Reveals Potential Regulatory Targets for MYCN

**DOI:** 10.3389/fonc.2021.633579

**Published:** 2021-04-21

**Authors:** Chunyu Pan, Yuyan Zhu, Meng Yu, Yongkang Zhao, Changsheng Zhang, Xizhe Zhang, Yang Yao

**Affiliations:** ^1^ Northeastern University, Shenyang, China; ^2^ Joint Laboratory of Artificial Intelligence and Precision Medicine of China Medical University and Northeastern University, Shenyang, China; ^3^ Early Intervention Unit, Department of Psychiatry, Affiliated Nanjing Brain Hospital, Nanjing Medical University, Nanjing, China; ^4^ Department of Urology, The First Hospital of China Medical University, Shenyang, China; ^5^ Department of Reproductive Biology and Transgenic Animal, China Medical University, Shenyang, China; ^6^ National Institute of Health and Medical Big Data, China Medical University, Shenyang, China; ^7^ School of Biomedical Engineering and Informatics, Nanjing Medical University, Nanjing, China; ^8^ Department of Physiology, Shenyang Medical College, Shenyang, China

**Keywords:** PPI network, MYCN, potential targets, network controllability, EGFR

## Abstract

**Background:**

MYCN is an oncogenic transcription factor of the MYC family and plays an important role in the formation of tissues and organs during development before birth. Due to the difficulty in drugging MYCN directly, revealing the molecules in MYCN regulatory networks will help to identify effective therapeutic targets.

**Methods:**

We utilized network controllability theory, a recent developed powerful tool, to identify the potential drug target around MYCN based on Protein-Protein interaction network of MYCN. First, we constructed a Protein-Protein interaction network of MYCN based on public databases. Second, network control analysis was applied on network to identify driver genes and indispensable genes of the MYCN regulatory network. Finally, we developed a novel integrated approach to identify potential drug targets for regulating the function of the MYCN regulatory network.

**Results:**

We constructed an MYCN regulatory network that has 79 genes and 129 interactions. Based on network controllability theory, we analyzed driver genes which capable to fully control the network. We found 10 indispensable genes whose alternation will significantly change the regulatory pathways of the MYCN network. We evaluated the stability and correlation analysis of these genes and found EGFR may be the potential drug target which closely associated with MYCN.

**Conclusion:**

Together, our findings indicate that EGFR plays an important role in the regulatory network and pathways of MYCN and therefore may represent an attractive therapeutic target for cancer treatment.

## Introduction

The MYC proto-oncogene family consists of three paralogs: c-MYC, MYCN, and MYCL (1, 2). Abnormal MYC regulation can lead to increased cell proliferation and growth, MYC family members are the dysregulation of MYC family is common in cancer (2). The MYCN cancer gene in the MYC family is a structurally and functionally similar fragment of MYC discovered by *Schwab* (3) in 1983. It acts to promote cell proliferation, and inhibit cell differentiation, apoptosis, or programmed cell death (4–6). Existing researches suggest that MYCN plays a key role in cell proliferation and cell growth during embryonic development (7) and it is associated with a number of childhood-onset tumors, including neuroblastoma, medulloblastoma, rhabdomyosarcoma, glioblastoma multiform, retinoblastoma, astrocytoma, hematologic malignancies, and small-cell lung cancer (8, 9), as well as some adult cancers such as prostate and lung cancer (10, 11). Despite the proven importance of MYCN, which has very promising therapeutic potential, how to directly target MYCN remains an open question. There is no better method to target MYCN directly in existing research (9), but we can still target MYCN indirectly by targeting molecules that interact directly with MYCN to control MYCN activity (9, 12–19). Thus, the problem of targeting MYCN can be translated into the study of the MYCN regulatory network of its interactions.

Recently, network controllability theory has made remarkable achievements in analyzing biological networks, such as Protein-Protein Interaction (PPI) network (20–24), brain network (25, 26) and disease-related networks (27, 28). *Ryouji* (20) applied network controllability theory on breast cancer gene expression networks, and designed a novel method to identify a set of critical control proteins that uniquely and structurally control the entire proteome. *Wu* (29) determined minimum dominating sets of proteins (MDSets) in human and yeast protein interaction networks and found that MDSet proteins were enriched with essential, cancer-related, and virus-targeted genes. *Guo* (30) developed an algorithm for identifying steering nodes to a gene regulatory network related to type 1 diabetes and they found that FASLG and CD80 are steering nodes for controlling the target nodes related to type 1 diabetes and supported by wet experiments.

In the view of control theory, drug targets in a biological network can be interpreted as a steering node. By applying an extra signal to this set of guide nodes, the network is expected to be steered to the desired state. In other words, for a biological system with an abnormal state, if some biomolecules affect other biomolecules by extra perturbations and steer the system towards a healthy state, these perturbed biomolecules can be considered potential drug targets. Thus, the problem of identifying drug targets can be mapped to the problem of finding a set of steering nodes in a network system. By applying a control signal to these nodes, the states of the network are expected to transition between the healthy state and the disease state.

Here, we utilized network controllability theory (31–36) to analyze the protein-protein interaction (PPI) network of MYCN. We identified possible potential drug targets of the MYCN regulatory network and evaluated the importance of these potential targets with several existing databases. The results showed that network controllability theory may provide new ideas to reveal the function of MYCN and target MCYN, which is of great importance and application prospect.

## Methods

### Network Controllability

Consider a linear time-invariant networked system, the dynamics of the process can be described as follows:

(1) $$\frac{dx\left(t\right)}{dt}=Ax\left(t\right)+Bu\left(t\right)$$

Where vector *x*(*t*) = (*x*_1_(*t*),…,x_N_(*t*))*^T^* represents the system state vector of N nodes at time t; matrix A is a state parameter describing the components of the system; matrix B of N*M(M≥ N) is the input matrix from which the controlled node is identified by the external controller. Vector *u*(*t*)=(*u*_1_(*t*),…,*u_M_*(*t*))*^T^* represents the input vector of M nodes at the time t and the controller uses the input vector *u*(*t*) to control the entire system and a single control signal *u_i_*(*t*) can typically drive multiple nodes.

According to the *Kalman* rank condition (31, 37):

$$\text{C}=\left(\text{B},A\text{B},{\text{A}}^{2}\text{B},\ldots ,A^{N-1}\text{B}\right)\text{rank}\left(\text{C}\right)=\text{N}$$

**Figure 1:**
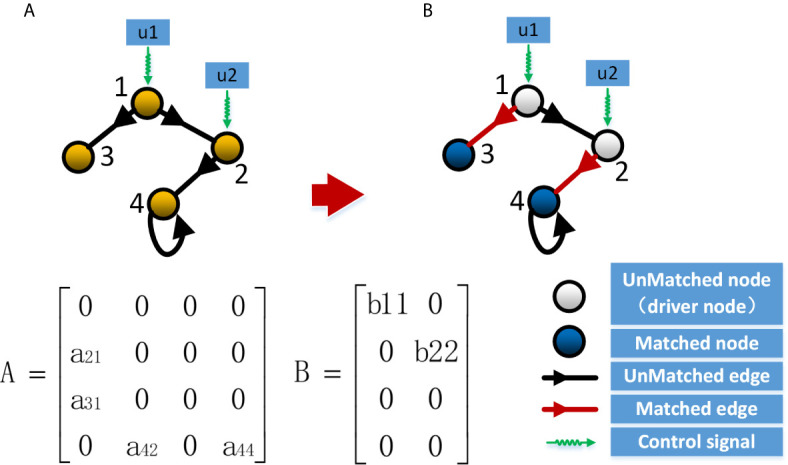
Control of the network system. **(A)** Controllability of a network through the controllability matrix; **(B)** Controllability of a network through the maximum matching.

The system is controllable if and only if the N*NM matrix C=(B,AB,*A* ^2^B,…,*A^N^* ^–1^B) is full rank, and the system can drive any initial state to any final state in a finite time. Based on this theory, *Lin *(33) proposed the theory of structural controllability, in which the state matrix A and the control matrix B can be regarded as a structured matrix, and if there are matrices A and B with non-zero weights that make the *Kalman* criterion hold, then for the way of combining different weights in matrices A and B, the system is almost always controllable except for the all-zero state and some special cases. On this basis, researchers in the field of network control (32, 34) have transformed the problem of least external input to a directed network into a problem of calculating the maximum matching for that network, as shown in **Figure 1**. For a directed network, a maximum matching is a set of maximal edges that do not share the starting and ending node, while nodes that do not have matching edges pointing to them are driver nodes. In contrast, the driver nodes computed by maximum matching is called minimum set of driver nodes (MDS). Since the maximum matching is often not unique for the same network, it is often possible to obtain multiple different MDS for the same network (38–41). In this case, we can analyze the nodes in different MDS and thus assess the importance of the nodes.

### Node Classification Based on Network Controllability

**Figure 2:**
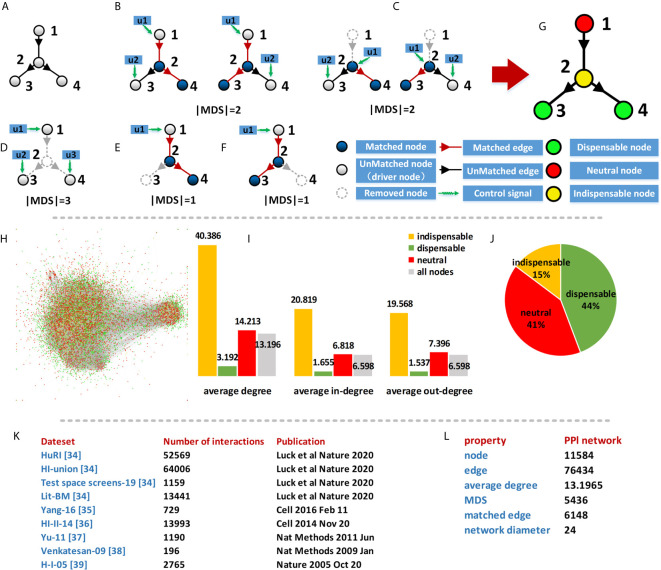
Characterizing of the PPI network. **(A)** A simple network; **(B)** Two different maximum matching of **(A)**; **(C–F)** The size of MDS after different node removed; **(G)** Classification results of **(A)**; **(H)** Classification results of PPI network; **(I)** Average degree of different type nodes in PPI network; **(J)** Percentages of different types in PPI network; **(K)** Data source of PPI network; **(L)** Basic property of PPI network.

This method measures the nodes in different MDS and considers the importance of the nodes in the whole network. For a network, MDS can be obtained by using the maximum matching method (34) and the type of node can be determined by the size of MDS after this node removing from the network. A node is indispensable if the size of MDS decreases after removing the node from the network. A node is dispensable if the size of MDS increases after removing the node from the network. A node is neutral if the size of MDS do not change after removing the node from the network. The simple network (**Figure 2A**) has two different maximum matching (**Figure 2B**), and the size of original MDS is 2. The size of the MDS will change when the nodes in this network are removed and the size of MDS after different nodes removed are shown in **Figures 2C–F**.

**Figure 3:**
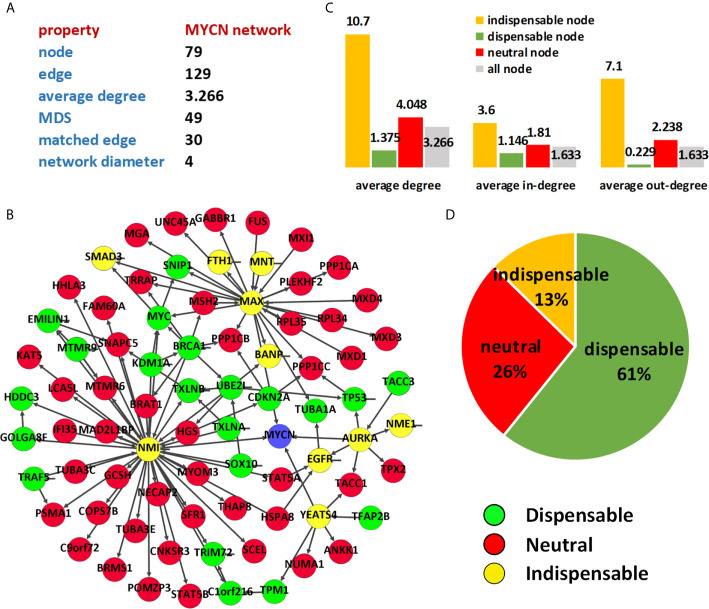
Characterizing of MYCN regulatory network. **(A)** Topological statistics of MYCN regulatory network; **(B)** Node classification of MYCN network; **(C)** Average degree of different type of nodes; **(D)** Percentages of different types in MYCN network.

In this simple network, the removal of node 1 does not change the MDS size of the network, as defined in the classification that node 1 is a neutral node. While the removal of node 2 increases the MDS size, and node 2 is an indispensable node. Similarly, node 3 and node 4 are dispensable nodes. The classification result of MYCN regulatory network is shown in **Figure 3B**.

### Source of Data Sets

The Cancer Genome atlas (TCGA, https://tcga-data.nci.nih.gov/tcga), a project initiated jointly by the National Cancer Institute (NCI) and the National Genome Research Institute (NHGRI). Utilize large scale sequencing based genomic analysis techniques to finalize a complete set of mapping associated with all cancer genomic alterations. To date, TCGA has been tested in over 10,000 human samples with whole cancers. We selected PanCancer Atlas Studies as our data set from TCGA for validating the results of the method, which included 32 different cancers with 10,967 samples. Survival analysis is provided by Cbioportal (www.cbioportal.org), it supports the use of custom data and provides researchers with an interactive interface to discover associations between genetic alterations and the clinic, and the data source for Cbioportal is TCGA. Co-expression and pathway analysis is also provided by Cbioportal, whose pathway data are provided by TCGA research and the TCGA PanCanAtlas project (42–50). These pathways have been rigorously extrapolated and validated and are published, which is of great biological significance and very important for the analysis of disease or gene interaction mechanisms.

Data sets of drug targets provided by *Behan* et al.’s work (51), they used genome-scale CRISPR–Cas9 screens in 324 human cancer cell lines from 30 cancer types and developed a data-driven framework to prioritize candidates for cancer therapeutics.

## Results

### Control Analysis of Human Protein-Protein Interaction Network

Consider a Protein-Protein interactions (PPI) network, a node of the network represents a protein and the interactions between proteins are the edges of the network. We used human binary protein interactions (HuRI) (52), a Protein interaction database which is the largest human protein interactome data to date. The protein-protein interaction in the network is of paramount importance both for understanding the underlying biological processes and for understanding disease occurrence. In addition, we have combined the protein-protein interactions provided by other databases (53–57) to form a more comprehensive network. The specific data sources are shown in **Figure 2A**.

The result of the PPI network consists of 11,584 proteins and 76,434 interactions. The average degree of the network is 13.2 and the diameter of the network is 24. To analyze the control properties of the PPI network, we used the maximum matching method to compute the Minimum Driver nodes Set (MDS) in the network. Although the MDSs are not unique for the PPI network, but the size of all MDSs is same and determined by the network topology. In the PPI network, there are 5436 (46.93%) driver proteins which composed of the MDS of the PPI network. It means that to fully control the PPI network, we need to control nearly half of the proteins in the network. Therefore, the MDS did not provide much information for identifying potential drug target of the network.

Furthermore, We used a control classification method (21) to divide the proteins into three types: indispensable, dispensable, and neutral proteins. This node classification is based on the size changes of MDS after removing the node from the network. A node is indispensable if the size of MDS decreases after removing the node from the network. A node is dispensable if the size of MDS increases after removing the node from the network. A node is neutral if the size of MDS do not change after removing the node from the network. An example network is shown in **Figure 2**. For the PPI network, a total of 1710 (15%) proteins are indispensable, 5218 (44%) proteins are dispensable nodes and 4749 (41%) proteins are neutral. We found the average degree of the indispensable nodes is much higher than the other class nodes, which means the selected indispensable proteins have more interactions and are more closely related to other molecules than the other proteins in the network.

### Control Analysis of MYCN Sub-Network

To find potential drug target of MYCN, we extracted the second-order egocentric network of MYCN from the PPI network. The MYCN-egocentric network includes the neighbor nodes that interact directly with MYCN and the neighbor nodes that interact with the neighbors of MYCN. We used the second-order egocentric network to analyze the MYCN network because the goal of our analysis is to find molecules that can be targeted among the direct or indirect interactions of MYCN, and the nodes we selected should not be too far away from MYCN. **Figure 3** shows the result of control analysis of MYCN network. The network consists of 79 nodes and 129 edges and the size of MDS of MYCN network is 49 (62.03%). The number of matching edges is 30 (23.26%) and the network diameter is 4.

By using the node classification method (21) based on controllability analysis, we computed the control types of the proteins in the MYCN network. As the same as the PPI network, the average degree of the indispensable nodes is much higher than the other type nodes in MYCN regulation network (**Figure 3C**). However, the value of average degree is not involved in the processing of the classification and the phenomenon is not accidental or biased. For all the nodes in the MYCN regulatory network, we found 10 (13%) nodes are indispensable, 21 (26%) nodes are neutral nodes and 48 (61%) nodes are dispensable. **Table 1** showed the indispensable proteins and their topological properties and associated diseases. Meanwhile, among these 10 indispensable nodes, MAX, AURKA, YEATS4, and NMI are the nodes directly associated with MYCN, these proteins are present in the first-order egocentric network of MYCN and have close interactions with MYCN.

**Table 1:** Indispensable genes in MYCN regulatory network.

symbol	Full name	Indegree	Outdegree	Diseases associated
AURKA (58)	Aurora Kinase A	1	6	Colorectal Cancer/Colorectal Adenocarcinoma.
BANP (59)	BTG3 Associated Nuclear Protein	3	1	Keratoconus/Brittle Cornea Syndrome 2
EGFR (60)	Epidermal Growth Factor Receptor	4	2	Inflammatory Skin/Bowel Disease, Neonatal, 2/Lung Cancer
FTH1 (61)	Ferritin Heavy Chain 1	2	1	Hemochromatosis/Type 5 and Superficial Siderosis Of The Central Nervous System
MAX (62)	MYC Associated Factor X	8	18	Pheochromocytoma/Hereditary Paraganglioma-Pheochromocytoma Syndromes
MNT (63)	MAX Network Transcriptional Repressor	1	2	Tetanus Neonatorum/Mixed Type Thymoma
NME1 (64)	NME/NM23 Nucleoside Diphosphate Kinase 1	2	1	Anal Canal Carcinoma/Larynx Cancer
NMI (65, 66)	N-Myc And STAT Interactor	11	34	–
SMAD3 (67)	SMAD Family Member 3	3	1	Loeys-Dietz Syndrome 3/Familial Thoracic Aortic Aneurysm And Aortic Dissection
YEATS4 (68)	YEATS Domain Containing 4	1	5	Cellular Myxoid Liposarcoma/Pleomorphic Liposarcoma

### Functional Analysis of Indispensable Proteins

**Figure 4:**
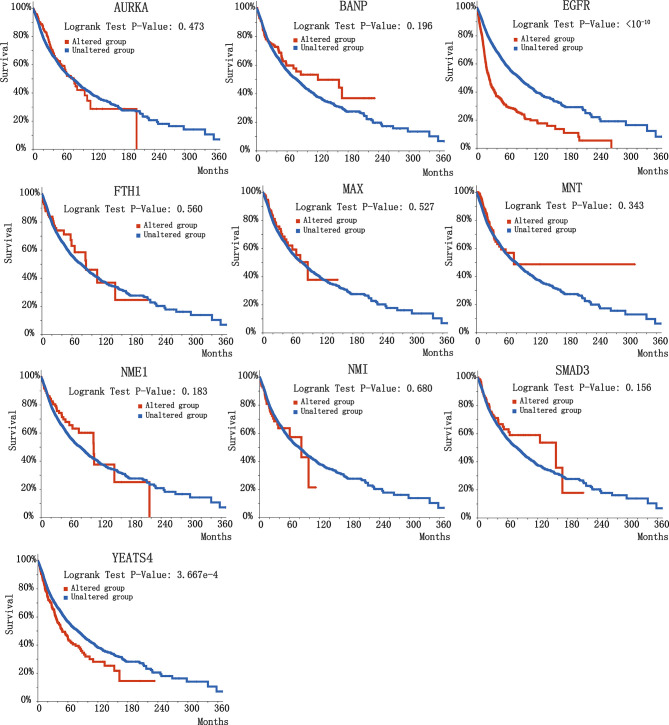
Survival curve of 10 indispensable genes. 10 plots correspond to different indispensable genes, here we chose overall survival data rather than disease-specific survival data.

To further investigate the biological significance of indispensable genes in the MYCN network, we perform survival analysis of indispensable genes base on the clinical data of The Cancer Genome Atlas (TCGA) (69) included 32 different cancers with 10,967 samples. Here we used overall survival without disease-specific for a gene, it can eliminate the survival differences in certain diseases. By plotting the relationship between survival months and surviving percentage, can obtain the differences in survival for altered group and unaltered group. **Figure 4** showed the clinical survival of 10 indispensable genes. Among the ten indispensable genes, EGFR and YEATS4 had a significant difference between the altered group and the unaltered group, which suggested that the mutation of these two genes will significantly change the survival of patients. Clinical samples and median survival Months are shown in **Table 2**. Considering the differences in disease grade and treatment strategy, we also divided the sample into multiple groups for statistical analysis (**Supplement 2**).

**Table 2:** Clinical samples of indispensable genes.

Id	Name	Type	Number of Cases, Total	Number of Cases, Deceased	Median Months Overall
**1**	**AURKA**	**Altered group**	233	77	80.74
		**Unaltered group**	10569	3437	78.97
**2**	**BANP**	**Altered group**	237	69	120.62
		**Unaltered group**	10565	3445	78.67
**3**	**EGFR**	**Altered group**	812	460	24.3
		**Unaltered group**	9990	3054	85.08
**4**	**FTH1**	**Altered group**	87	27	88.11
		**Unaltered group**	10715	3487	78.9
**5**	**MAX**	**Altered group**	99	32	89.72
		**Unaltered group**	10703	3482	78.97
**6**	**MNT**	**Altered group**	147	41	74.93
		**Unaltered group**	10655	3473	78.97
**7**	**NME1**	**Altered group**	153	42	105.04
		**Unaltered group**	10649	3472	78.67
**8**	**NMI**	**Altered group**	102	30	83.57
		**Unaltered group**	10700	3484	78.97
**9**	**SMAD3 **	**Altered group**	175	48	156.49
		**Unaltered group**	10627	3466	78.67
**10**	**YEATS4**	**Altered group**	318	141	49.05
		**Unaltered group**	10483	3373	80.48

**Figure 5:**
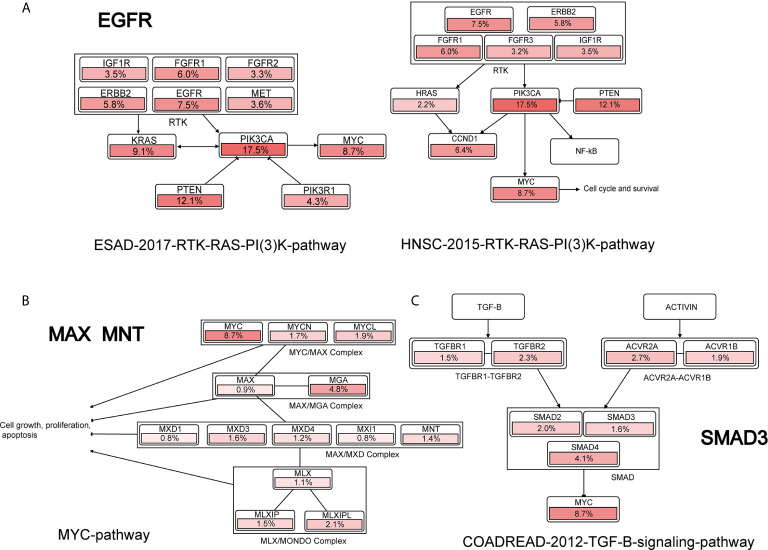
Cancer pathway of indispensable genes. **(A–C)** are the pathways that EGFR, MAX, MNT, SMAD3 associated with MYCN or MYC, respectively.

Furthermore, we performed pathway analysis for the indispensable genes (42–50) based on Cbioportal (70). We found that EGFR, MAX, MNT and SMAD3 are associations with MYCN or MYC family in several pathways, as shown in **Figure 5**. EGFR was indirectly associated with MYC activity in ESAD-2017-RTK-RAS-PI(3)K-pathway and HNSC-2015-RTK-RAS-PI(3)K-pathway by PIK3CA. MAX and MNT are correlated with MYCN in the MYC-pathway, where MAX and MYCN form the MYC/MAX complex, and MNT associated with the MAX/MGA complex. The pathway analysis shows that the indispensable genes computed by the network controllability theory are precise and are directly or indirectly associated with MYCN in different pathways.

Finally, we analyzed the indispensable node that are targeted by the drugs. Based on the database of drug targets in 324 human cancer cell lines from 30 cancer types (51), we found that EGFR is an anti-cancer target in Squamous Cell Lung Carcinoma, Lung Adenocarcinoma, Oral Cavity Carcinoma, Ovarian Carcinoma, Head and Neck Carcinoma and Esophagus. It has a high priority and has a class B biomarker, making it a more desirable target. EGFR has at least one drug that has been developed for the cancer type in which the target was identified as a priority. In relation to our research of the MYCN regulatory network, EGFR may be the potential drug target which closely associated with MYCN.

Overall, based on the survival analysis, cancer pathway and drug targets analysis of indispensable genes, it is clear that the indispensable genes have a significant role in the MYCN regulatory network. The indispensable genes are directly associated with cancers, especially EGFR, MAX, MNT, SMAD3. EGFR is also a drug target that has already been developed and is considered to be the most promising potential target in the MYCN regulatory network.

### Indispensable Proteins in Brain Lower Grade Glioma

**Figure 6:**
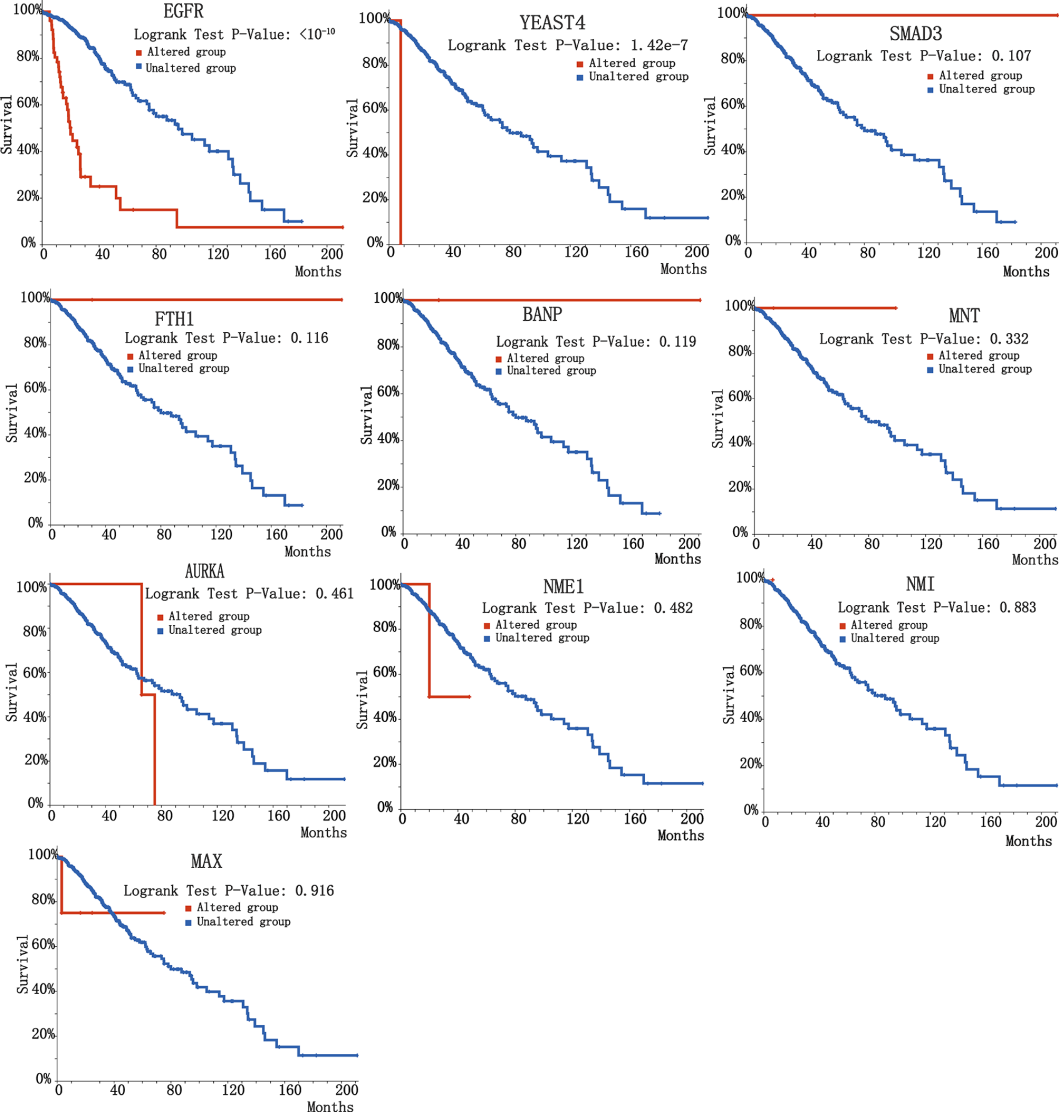
Survival curve of 10 indispensable genes of Brain Lower Grade Glioma. 10 plots are the survival curves of different indispensable genes and Brain Lower Grade Glioma respectively.

To further validate the biological significance of indispensable genes, in this section, we verified the effectiveness of our results with the specific-diseases. For the choice of specific-diseases, we should select a disease that is associated with MYCN, to analyze the survival of indispensable genes and the co-expression relationship with MYCN. Due to MYCN plays a key role in cell proliferation and cell growth during embryonic development (7) and it is often associated with a number of childhood-onset tumors, here we combined Brain Lower Grade Glioma to show the results of analysis. The survival curves for indispensable genes for Brain Lower Grade Glioma are shown in **Figure 6**. And the co-expression correlation between indispensable genes and MYCN of Brain Lower Grade Glioma are shown in **Table 3**. We found that BANP, NME1, YEATS4, and EGFR, have relatively significant Spearman’s Correlation with MYCN. Among them, YEATS4 has been shown in existing studies to have a direct interaction with MYCN (53–57). Although there are no direct association between three other genes and MYCN in existing studies, from the co-expression of Brain Lower Grade Glioma, it is possible that had correlation between them.

**Table 3:** Co-expression correlation between indispensable genes and MYCN of Brain Lower Grade Glioma.

Name	Spearman’s Correlation	p_value	q_value
EGFR	0.274	2.83E-10	1.29E-09
AURKA	0.137	1.913E-3	3.532E-3
BANP	0.26	2.25E-09	9.14E-09
FTH1	-0.282	7.77E-11	3.78E-10
MAX	0.085	0.054	0.0775
MNT	0.161	2.418E-4	5.084E-4
NME1	0.29	1.96E-11	1.02E-10
NMI	-0.3	3.72E-12	2.11E-11
SMAD3	0.0736	0.0954	0.13
YEATS4	0.361	3.02E-17	3.14E-16

## Discussion

MYCN plays an important role in many diseases and cancers (2, 7–11), in-depth understanding of the role of MYCN has a great significance and application prospect. However, MYCN is difficult to directly target and design therapeutic strategies in existing research (9). Therefore, we hope to find potential targets around the MYCN regulatory network and regulate MYCN indirectly by controlling the potential targets. By using network controllability method (21), we found ten indispensable genes in the MYCN regulatory network. Through the pathway, survival, drug target analysis, we found that the indispensable genes, especially EGFR, play an important role in MYCN regulatory networks.

To validate the biological significance of indispensable genes, especially EGFR, we calculated the correlation between the 10 indispensable genes and MYCN using the TCGA dataset (**Supplement 1**). For the 33 cancers proposed by TCGA, we analyzed spearman’s correlation, p-value (2-sided t-test), and q-value (Benjamini-Hochberg FDR correction) of MYCN with indispensable genes in expression in different diseases sequentially. Our core target EGFR had significant positive correlation results in Thymoma, Kidney Chromophobe, Diffuse Large B-Cell Lymphoma, Brain Lower Grade Glioma, and Skin Cutaneous Melanoma. All other indispensable genes also had a significant co-expression results with MYCN in specific diseases, this is concur with the results of existing studies. For the ten potential targets we obtained, MAX, AURKA, YEATS4 and NMI are directly associated with MYCN. MAX and AURKA in particular have been rigorously argued to be tightly associated with MYCN activity (71). For the other 6 potential targets, they are indirectly connected to MYCN. Although current research of these genes hasn’t a direct interaction with MYCN, in the theory of network control when this type of node changes, it can alter the features of network and affect the state of MYCN result in indirectly target MYCN. Among them, EGFR, MNT, and SMAD3 are all directly or indirectly associated with the MYCN or MYC families in different pathway. EGFR, in particular, is not only significantly different between the altered and unaltered groups in clinical survival data, but also a molecule that can already be drug-targeted (51).

As the driving gene of many kinds of tumors, EGFR plays an important role in promoting the malignant progression of tumors (60). Its role in non-small cell lung cancer, glioblastoma and basal-like breast cancers has spurred many research and drug development efforts. Tyrosine kinase inhibitors have shown efficacy in EGFR amplified tumors, most notably gefitinib and erlotinib. But the mutations in EGFR have been shown to confer resistance to these drugs, particularly the variant T790M, which has been functionally characterized as a resistance marker for both of these drugs. The later generation TKI’s have seen some success in treating these resistant cases, and targeted sequencing of the EGFR locus has become a common practice in treatment of non-small cell lung cancer (72–74). Therefore, we consider EGFR to be the most promising potential target among these indispensable genes (**Supplement 2**).

Meanwhile, referring to the biological properties of MYCN (7, 75) (**Supplement 2**), we selected Brain Lower Grade Glioma to validating indispensable genes. Among them, BANP, NME1, YEATS4, and EGFR, have relatively significant Spearman’s Correlation with MYCN. It is worth noting that NMI has a high negative correlation with MYCN. Due to the algorithm views the biological network as an abstract network structure in isolation from the specific biological constraints, this algorithm without specific biological constraints is able to filter out genes with high correlation (positive and negative), not just positive correlation. And NMI as an interactor of MYCN, has a high absolute value of correlation with MYCN in the network, which is consistent with the algorithm results. For EGFR, which we considered the most potentially target, there were more significant results in Brain Lower Grade Glioma, both in the co-expression and survival.

Each cancer is extremely complex and different networks will come with different results. In this study, we chose pan-cancer data to construct a more comprehensive network to predict potential targets for MYCN in terms of overall relationships, and finally verified the effect of indispensable genes combined with specific-diseases. The theory of network controllability bring a new view and theoretical framework to the analysis of regulatory networks. However, the composition of nodes and edges will impact the accuracy of the results. Therefore, it is still a challenge to accurate construction of the initial network and find the exact target network from a large amount of data and specific-diseases. This is a new methodological trying to identify potential targets, and after the network control framework analysis, how to design wet experiments to further verify the analysis results is also one of our subsequent concerns.

Overall, the method of network controllability in this paper is able to screen potential targets against MYCN and our findings indicate that EGFR plays an important role in the MYCN regulatory network. In the future, experimental evidence to support the above regulatory relationship will be further provided through *in vitro* and *in vivo* experimental systems, so as to promote the identification and discovery of potential new regulatory targets.

## Data Availability Statement

The original contributions presented in the study are included in the article/**Supplementary Material**. Further inquiries can be directed to the corresponding authors.

## Ethics Statement

The studies involving human participants were reviewed and approved by TCGA Ethics & Policies and were originally published by the National Cancer Institute. The patients/participants provided their written informed consent to participate in this study. Written informed consent was obtained from the individual(s) for the publication of any potentially identifiable images or data included in this article.

## Author Contributions

XZZ is the lead author. XZZ, YYZ, YKZ and YY conceived the study and revised the manuscript. CYP and CSZ performed data analysis and interpretation and drafted the manuscript. MY searched the databases and acquired the data. All authors contributed substantially to the preparation of the manuscript.

## Funding

This work was supported by the Fundamental Research Funds for the Central Universities (N2017013, N2017014), National Natural Science Foundation of China (U1908212, 81672523, 81472404, 81472403, 81272834 and 31000572), 2018 Support Plan for innovative talents in Colleges and Universities of Liaoning Province, 2018 “million talents Project” funded Project of Liaoning Province, 2019 Key R & D Projects of Shenyang.

## Conflict of Interest

The authors declare that the research was conducted in the absence of any commercial or financial relationships that could be construed as a potential conflict of interest.
