# Supplementary material for: Control Analysis of Protein-Protein Interaction Network Reveals Potential Regulatory Targets for MYCN
Source: Front Oncol. 2021 Apr 21;11:633579. doi: 10.3389/fonc.2021.633579 (PMC8096904; doi:10.3389/fonc.2021.633579)
Supplement: Supplementary file 1 [file DataSheet_1.docx]

Supplementary Material

### Grade and treatment strategy

Based on the overall survival analysis, we also considered the impact of different disease grade and treatment strategies. The distribution of radiation therapy and disease grade among the different groups is illustrated in Figure 1. For each group, the results of the statistical tests are presented in supplement 1. As well, in supplement 1, the clinical information of all sample of LGG in TCGA has been included.


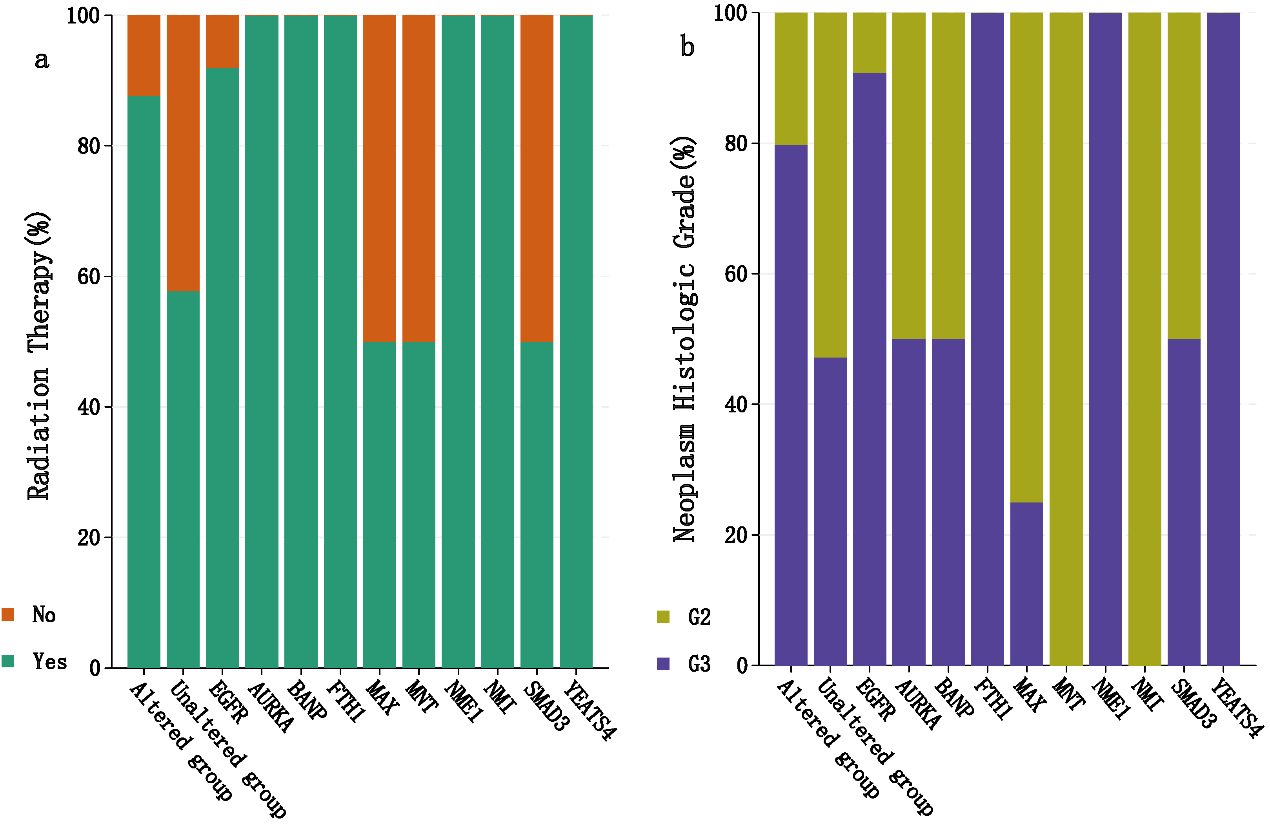


**Supplementary Figure 1:** Disease grade and treatment strategy in the sample. (a) Percentage of radiation therapy in different groups; (b) Percentage of disease grade in different groups.

#### MYCN in Neuroblastoma

Within two years of MYCN’s discovery in neuroblastoma, amplification of MYCN was shown to correlate with poor prognosis in patients [1], a biomarker that is still used today to stratify risk. Mice with targeted misexpression of MYCN to the peripheral neural crest via the rat tyrosine hydroxylase (TH)-promoter developed neu-roblastoma, establishing that misexpression of MYCN in migrating neural crest cells can initiate this disease [2]. Tumors in these mice had a prolonged latency and showed recurrent chromosomal copy number abnormalities, suggesting that genetic mutations in addition to misexpressed MYCN were required to promote neuroblast transformation. This requirement for additional mutations was supported by the fact that loss of tumor suppressors neurofibromin1 or retinoblastoma1 (Rb), when combined with misex pression of MYCN, resulted in reduced latency and increased penetrance for tumors [3].

#### Pharmacological EGFR inhibitors

Small molecule TKIs is the mostly pharmacological inhibitors, targeting EGFR have been extensively tested in preclinical glioma models. Similar to their practice with other tumor entities such as lung carcinomas, for which these drugs are well established in clinical practice, most investigators used erlotinib or gefitinib to interfere with EGFR signaling. The EGFR-blocking activity of erlotinib and gefitinib largely depends on the presence of mutations in exons 19 and 21 of the TK domain. These mutations are commonly found in lung cancer and other tumor entities and have led to the approval of several EGFR inhibitors. However, these “sensitizing” mutations are virtually absent in glioblastomas, which may partially explain the lack of activity of standard TKIs in this disease [4-8].

#### Antibodies against EGFR

Antibodies directed against EGFR—with cetuximab, nimotuzumab, and panitumumab as the most prominent candidates—were also investigated for their antiglioma activity in vitro andin vivo. Antibodies may exert their effect by preventing the binding of EGFR ligands to the receptor. Furthermore, antibody binding may result in receptor internalization and degradation[9]. Although antibodies to EGFR have been approved for other cancer types, such as cetuximab for the treatment of Kirsten rat sarcoma viral oncogene homolog wild-type colon cancer, their use against intracranial neoplasms such as glioblastoma represents a challenge due to the presence of the blood– brain barrier, which may preclude the penetration of the antibody to all parts of the tumor. However, small molecule EGFR inhibitors such as erlotinib and gefitinib also did not markedly inhibit EGFR phosphorylation in vivo[10]. Accordingly, poor tumor perfusion or the blood – brain barrier may represent an important “resistance factor” that limits the activity of EGFR-targeting drugs in the brain[4].

## Reference

[1] Brodeur, G. M., Seeger, R. C., Schwab, M., Varmus, H. E. and Bishop, J. M. J. S. Amplification of N-myc in untreated human neuroblastomas correlates with advanced disease stage, 224 (

[2] Weiss, W. A., Aldape, K., Mohapatra, G., Feuerstein, B. G. and Bishop, J. M. J. E. J. Targeted expression of MYCN causes neuroblastoma in transgenic mice, 16, 11 (2014), 2985-2995.

[3] Huang, M. and Weiss, W. A. J. C. S. H. p. i. m. Neuroblastoma and MYCN, 3, 10 (2013), a014415.

[4] Roth, P. and Weller, M. J. N.-o. Challenges to targeting epidermal growth factor receptor in glioblastoma: escape mechanisms and combinatorial treatment strategies (2014), viii14-19.

[5] Munoz, J. L., Rodriguez-Cruz, V., Greco, S. J., Ramkissoon, S. H., Ligon, K. L., et al. Temozolomide resistance in glioblastoma cells occurs partly through epidermal growth factor receptor-mediated induction of connexin 43, 5, 3 (2014), e1145.

[6] Marie, Y., Carpentier, A. F., Omuro, A. M. P., Sanson, M., Thillet, J., et al. EGFR tyrosine kinase domain mutations in human gliomas, 64, 8 (2005), 1444-1445.

[7] Mellinghoff, I. K., Wang, M. Y., Vivanco, I., Haas-Kogan, D. A. and Mischel, P. S. J. 新. Molecular determinants of the response of glioblastomas to EGFR kinase inhibitors, 353, 19 (2005), 2012-2024.

[8] Mischel, P. S., Iwanami, A., Reardon, D. A., Grommes, C., Gilbert, R. D., et al. Differential Sensitivity of Glioma- versus Lung Cancer–Specific EGFR Mutations to EGFR Kinase Inhibitors, 2, 5 (2012), 458-471.

[9] The Somatic Genomic Landscape of Glioblastoma %J Cell (2013).

[10] Weiner, L. M., Surana, R. and Wang, S. J. N. R. I. Monoclonal antibodies: versatile platforms for cancer immunotherapy
